# Risk Factors at Birth for Permanent Obstetric Brachial Plexus Injury and Associated Osseous Deformities

**DOI:** 10.5402/2012/307039

**Published:** 2012-02-01

**Authors:** Rahul K. Nath, Nirupama Kumar, Meera B. Avila, Devin K. Nath, Sonya E. Melcher, Mitchell G. Eichhorn, Chandra Somasundaram

**Affiliations:** Research Division, Texas Nerve and Paralysis Institute, Houston, TX 77030, USA

## Abstract

*Purpose*. To examine the most prevalent risk factors found in patients with permanent obstetric brachial plexus injury (OBPI) to identify better predictors of injury. *Methods*. A population-based study was performed on 241 OBPI patients who underwent surgical treatment at the Texas Nerve and Paralysis Institute. *Results*. Shoulder dystocia (97%) was the most prevalent risk factor. We found that 80% of the patients in this study were not macrosomic, and 43% weighed less than 4000 g at birth. The rate of instrument use was 41% , which is 4-fold higher than the 10% predicted for all vaginal deliveries in the United States. Posterior subluxation and glenoid version measurements in children with no finger movement at birth indicated a less severe shoulder deformity in comparison with those with finger movement. *Conclusions*. The average birth weight in this study was indistinguishable from the average birth weight reported for all brachial plexus injuries. Higher birth weight does not, therefore, affect the prognosis of brachial plexus injury. We found forceps/vacuum delivery to be an independent risk factor for OBPI, regardless of birth weight. Permanently injured patients with finger movement at birth develop more severe bony deformities of the shoulder than patients without finger movement.

## 1. Introduction

The incidence of obstetric brachial plexus injury (OBPI) is about 1.51 [1] per 1000 live births in the United States and reports vary from 0.38 [2] to 5.8 [3] per 1000 live births. Many of these injuries are transient; however, most of the OBPI patients never recover full function and develop permanent injuries [2, 4, 5]. In reports conducted by pediatricians and specialists, with follow-up times greater than 3 years, the reported proportion of injuries that remain permanent varies from 50 to 90% [6–8]. Risk factors for injury include shoulder dystocia, macrosomia (defined as birth weight greater than 4500 g [9–12]) instrument-assisted delivery, and downward traction of the fetal head [1, 7, 8]. Yet in a database search of over 11 million births, it was found that most children with neonatal brachial plexus palsy did not have known risk factors [1].

In obstetrics, presentation of shoulder dystocia is often emergent because the reported risk factors for its occurrence are not good predictors of it [13, 14]. Therefore we seek to examine the most prevalent risk factors found in a population of patients with permanent OBPI that necessitated surgical treatment to attempt to identify better predictors of injury and to elucidate the pathophysiology of OBPI.

Recently, studies have determined risk factors specifically for permanent brachial plexus injury including birth weight [15], delivery methods [16, 17], and maternal factors [16, 17]. Risk factors for permanent injury may also contribute to the secondary glenohumeral and scapular deformities that result from neonatal brachial plexus injury. It has already been shown that clavicular fractures at birth are associated with bony deformities of the glenohumeral joint [18]. Instrument-assisted delivery, birth weight, shoulder dystocia, and lack of finger movement at birth have not been evaluated for their association with shoulder deformity.

Patients with permanent obstetric brachial plexus injuries frequently develop bony deformities which are caused by muscle imbalance on the developing bony elements of the infant shoulder developing from the asymmetrical brachial plexus injury, with the upper plexus (C5-6) being injured more commonly than the lower (C8-T1) [19]. These secondary deformities, including internal rotator and adductor contractures, glenohumeral dysplasia, humeral head posterior subluxation or dislocation, and/or scapular elevation and rotation, cause major long-term morbidity requiring surgical correction to improve limb function. In studies that quantify obstetric brachial plexus deformities, the most common measurements are posterior subluxation of the humeral head, glenoid retroversion, scapular deformity (SHEAR), and glenoid shape from CT or MRI images [20–23].

This study will explore potential risk factors of permanent brachial plexus injury and determine if these risk factors can predict the development of osseous deformities.

## 2. Materials and Methods

During a 19-month period between August 2006 and April 2008, 249 patients from various locations in the United States, Asia, and Europe were surgically treated for the sequelae of permanent obstetric brachial plexus injury at our institute. 241 patients were selected for this study; of the 8 patients excluded, 4 patients were excluded due to insufficient data, and 4 patients sustained their injury during Caesarian section or breech delivery. All the patients in this study were injured severely enough to develop shoulder deformities that required surgical reconstruction. All of the patients in the study have had at least one reconstructive surgery related to the initial nerve injury. All surgeries were performed by the same surgeon (RKN), whose practice has focused on reconstructive surgery in this population for the past 12 years. There were 122 (51%) girls and 119 (49%) boys with an average age of 5.6 years at the time of visit, ranging from 5 months to 27 years.

Patients were evaluated with a physical exam which included a modified Mallet functional assessment. Information regarding instrument use during delivery, birth weight, and shoulder dystocia was obtained from the patient's parent or guardian during the initial evaluation. Instrument use was unknown for two patients.

**Figure 1:**
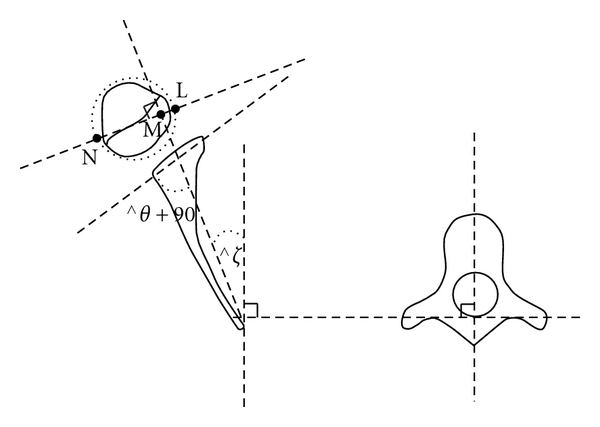
Schematic drawing showing the method of calculating glenoscapular angle (glenoid version *θ*), posterior subluxation of the humeral head (PHHA). The scapular line that connects the medial aspect of the scapula and the mid glenoid is drawn. A second line is drawn connecting the posterior and anterior margins of the glenoid. 90° are subtracted from the angle of the posterior medial quadrant deWned by these lines to determine the glenoid version *θ*. A line perpendicular to the scapular line is drawn, and the percentage of posterior subluxation is defined as the ratio of the distance from the scapular line to the anterior portion of the head to the diameter of the humeral head (LM/LN ×  100).

**Figure 2:**
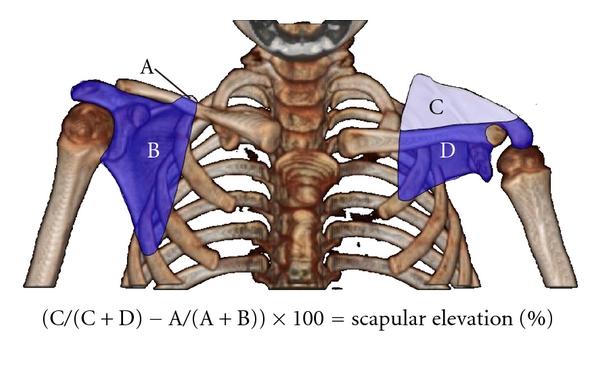
Measuring scapular elevation to quantitate the extent of the SHEAR deformity. A 3D-reconstruction of axial bilateral CT images rotated into the anterior view is used to measure scapular elevation. The area of each portion of both scapulas is measured as indicated (areas A-D). The area above the scapula is divided by the total scapular area and corrected for rotational artifacts by subtraction of the unaffected side from the affected side before multiplying by 100 to obtain percent elevation.

Presence of deformity was confirmed with analysis of CT and/or MRI scans obtained prior to surgical treatment. Posterior subluxation (measured as PHHA, percent humeral head anterior to the scapular line, see Figure 1), glenoid version (angle difference between the glenoid and a line 90° to scapular line), and SHEAR (scapular hypoplasia, elevation and rotation measured as percent scapula superior to the clavicle, see Figure 2) were measured from either CT or MRI scans as previously described [20, 21]. 

Measurements of PHHA and version were not available for 49 patients and SHEAR was not available for 89 patients. Increasingly severe deformity is associated with PHHA values decreasing from 50%, version values decreasing from 0°, and SHEAR values increasing from 0%. Glenoids were divided into 2 groups based on the presence (more deformed) or absence (less deformed) of a pseudoglenoid in CT or MRI images [23]. A pseudoglenoid is present when the glenoid is biconcave or shows a posterior concavity concentric with the humeral head in axial images.

A study population of transiently injured patients was not available. Therefore, data including the transiently injured population was gathered from published studies for comparison. National birth statistics were obtained from the Centers for Disease Control and Prevention website for the year 2003 [24]. Macrosomia was defined as >4,500 g [17, 25, 26] and appropriate for gestational age as <3,750 g [25].

Averages are reported as the mean ± one standard deviation and proportions are provided with 95% confidence intervals determined by the modified Wald method. The Mann-Whiney test was used for comparisons of continuous data. Categorical data and proportions were analyzed using Fisher's exact test. The proportion of injuries that remain permanent was pooled from multiple studies into two groups, studies conducted by obstetricians (*n* = 5) and studies conducted by pediatricians and specialists (*n* = 5). The two compiled proportions were then compared with Fisher's exact test. Normality of distribution was determined with the Shapiro-Wilk test. *P*  values < 0.05 were considered significant.

## 3. Results

### 3.1. Birth Weight

Our data showed that the average birth weight was 4085 ± 476 g (min 3005 g, max 5812 g) and birth weight was normally distributed (*P* = 0.73). Macrosomia was present in 20% (16–26%, 49/241) of the patients; 55% (48–61%, 132/241) weighed between 3750 and 4499 g at birth; 25% (20–31%, 60/241) weighed less than 3750 g at birth (see Table 1).

In macrosomic patients, glenoid version was significantly worse (*P* = 0.001) and posterior subluxation was not significantly different (*P* = 0.06), but lower when compared to patients below 4500 g (Table 2). The severity of the SHEAR deformity as determined by scapular elevation did not differ between these two groups (Table 2).

### 3.2. Shoulder Dystocia

The rate of documented shoulder dystocia among children with permanent brachial plexus injury was 97% (93–98%, Table 1). Birth weight was significantly higher in patients with documented shoulder dystocia (4100 ± 474 g) than those without documented shoulder dystocia (3634 ± 307 g; *P* = 0.005). Posterior subluxation and glenoid version were both worse in children with documented shoulder dystocia, but the difference was not significant because of the small number of patients in the group without documented shoulder dystocia (*n* = 8, Table 2).

### 3.3. Instrument-Assisted Delivery

Instruments (forceps, vacuum, or both) were used in 41% (35–47%) of the deliveries studied (Table 1). The average birth weight was indistinguishable for all instrument delivery categories (vacuum: 4013 ± 443 g, forceps: 3997 ± 424 g, both instruments: 4124 ± 661 g, no instruments: 4125 ± 467 g). The mean birth weight in instrument-assisted deliveries (4032 ± 489 g) was not significantly different from spontaneous deliveries (4125 ± 467 g, *P* = 0.11). The rate of instrument use decreased in higher birth weight categories (Table 1).

The SHEAR deformity was significantly worse (*P* = 0.04) in all instrument-assisted deliveries as compared to spontaneous deliveries. The significance was lost when forceps or vacuum extraction used alone was compared separately to spontaneous delivery (*P* = 0.17, 0.30, resp.) but was maintained when both instruments were used for the same delivery (*P* = 0.03). PHHA and version did not differ between instrument-assisted and spontaneous deliveries (*P* = 0.45, 0.79, resp. Table 2).

### 3.4. Finger Movement at Birth

No finger movement at birth was observed in 68% (61–73%) of the patients (Table 1). Mean birth weight among patients with no finger movement was 4089 ± 477 g; mean birth weight among those with finger movement was 4075 ± 478 g (*P* = 0.88). Lack of finger movement at birth was not associated with macrosomia, documented shoulder dystocia, or instrument use (*P* = 0.82, 0.47, and 0.91, resp. Table 2).

PHHA and version measurements in children with no finger movement at birth indicated a less severe shoulder deformity in comparison with those with finger movement at birth (*P* = 0.046, 0.001, resp.  Table 3). The glenoid fossa was also less deformed; a higher proportion of less severely deformed glenoids (non-pseudoglenoid) was observed in patients without finger movement (81%, 73–87%) than in those with finger movement (67%, 55–77%), although this difference was not significant (*P* = 0.055).

## 4. Discussion

Risk factors for sustaining an obstetric brachial plexus injury have been previously identified; however, the most pertinent risk factors, those associated specifically with permanent injury, have only recently been studied [3, 15–17, 27]. The proportion of injuries that remain permanent was thought to be less than 10% [28]; however, a review by Pondaag, et al. concluded that the most commonly reported recovery rates were based upon inadequate study methodologies [28]. In studies focusing on temporary versus permanent obstetric brachial plexus injury, the criteria for permanent injury are often not fully described, the follow-up periods are inadequate, or the end-stage evaluation is not performed by a brachial plexus specialists. Indeed, the proportion of injuries that remain permanent is significantly lower among studies conducted by obstetricians (13%, 10–17%, 55/419) [3, 16, 17, 27, 29] than pediatricians and orthopedic surgeons (51%, 43–58%, 86/170) [6–8, 30, 31] (*P* < 0.0001). For these reasons, data gathered from permanent injury in this study were contrasted with findings common to all brachial plexus palsies rather than data specific for transient palsies. Birth weight, shoulder dystocia, instrument use, and finger movement at birth were evaluated as potential risk factors for permanent injury and predictors of future osseous shoulder deformity.

The average permanent injury birth weight in this study was 4085 ± 476 g, which is lower than the average birth weight reported in the literature for brachial plexus injuries at 4265 ± 480 g [32], 4227 g [8], 4205 ± 608 g [17], and  4500 ± 625  [33]. Additionally, the rate of macrosomia in this study was 20% (16–26%) which is less than a previously reported rate for brachial plexus injuries at 29% [17] (20–38%). Among patients with documented shoulder dystocia in this study, the rate of macrosomia was 21% (16–27%, 49/233); lower than previous findings of Gherman et al., at 26% and 28%, for shoulder dystocia-associated brachial plexus injury [15, 34]. Thus, high birth weight is a risk factor for shoulder dystocia [35, 36] and brachial plexus injury in general [1, 37], but it is an unreliable predictor of permanent injury. This was further demonstrated by comparing the birth weight distributions of permanent brachial plexus injuries with all brachial plexus injuries (Figure 1).

**Figure 3:**
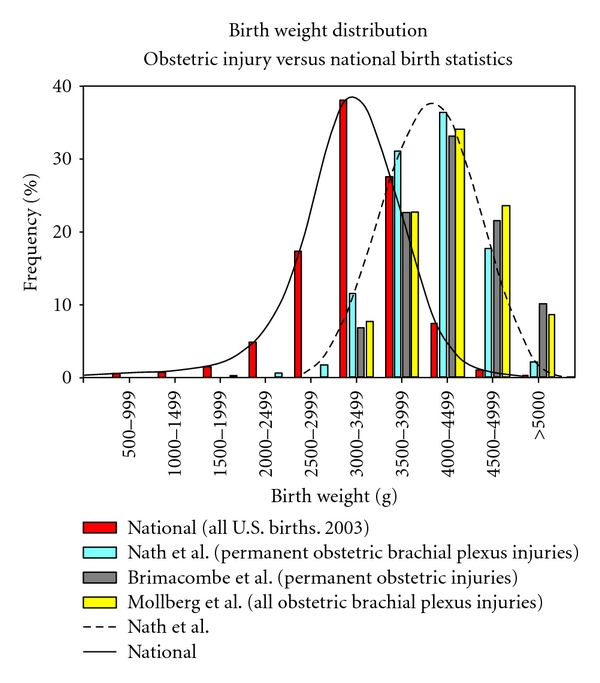
The birth weight distribution of permanent obstetric brachial plexus injury [21] and permanent obstetric injury (Brimacombe et al. [25]) compared to the distribution of birth weight among all brachial plexus injuries, which includes both transient and permanent injuries (Mollberg et al. [38]), and all births in the United States [39].

It is not implied that birth weights in permanent injury are similar to the normal population; birth weight among injured patients is higher than average, as demonstrated in Figure 3. Birth weight is not, however, associated with injury severity. Glenoid retroversion was significantly more severe in macrosomic patients, which suggests that although macrosomia cannot reliably predict permanent injury, macrosomia is associated with the development of a more severe glenohumeral deformity. Posterior subluxation was also more severe in macrosomic patients, although not significantly (Table 2). 

Permanent injury is not exclusive to large infants; 80% (74–84%) of the patients in this study were not macrosomic and 43% (104/241, 37–49%) weighed less than 4000 g at birth (Table 1). 52% (126/241, 46–58%) of the patients were below the estimated 90th percentile for birth weight at 40-week gestation and 11% (26/241, 7–15%) were below the 50th percentile [40]. Our findings indicate that nonmacrosomic fetuses frequently experience shoulder dystocia and develop permanent OBPI, despite the fact that macrosomia is said to be one of the primary indicators of permanent OBPI [38]. The average birth weight in this study was indistinguishable from the average birth weight reported for brachial plexus injuries. Higher birth weight does not, therefore, affect the prognosis of brachial plexus injury. Infants with birth weights normal for gestational age are still susceptible to severe permanent brachial plexus injuries.

It is important that delivering caregivers consider that nonmacrosomic babies may sustain injury to the brachial plexus. During the process of delivery, maneuvers causing the twisting and extension of the fetal head, which result in the stretching of the fetal neck, may be responsible for OBPI [41]. Gonik et al. report evidence, based on computational modeling of intrauterine forces, of increased brachial plexus stretch caused by lithotomy positioning during delivery, while acute flexion of the hips in the supine position (McRobert's maneuver) resulted in 53% less brachial plexus stretch [42]. Since the brachial plexus is the most complex peripheral neural unit, every effort needs to be taken to ensure that the brachial plexus is not injured during delivery [21].

In our study the rate of instrument use was 41% (35–48%), which is 4-fold higher than the 10% predicted for all vaginal deliveries in the United States [25, 43] (Table 1). Instrument use in our permanently injured patient group is in agreement with rates previously reported for all brachial plexus injuries in Chauhan et al. (45%) [27] and Backe et al. (40%) [17]. Instrument use is therefore equally represented in both temporary and permanent brachial plexus injuries. As shown in Table 1, the rate of instrument use consistently decreased with increasing birth weight from 47% (<3750 g; 35%–60%) to 33% (>4500 g; 21%–41%). This finding, also observed by Brimacombe et al., is contrary to a report published in the American College of Obstetricians and Gynecologists Technical Bulletin that instrument use is higher in the delivery of macrosomic fetuses [25, 44]. Therefore, we found forceps/vacuum delivery to be an independent risk factor for OBPI, regardless of birth weight. This finding points to the fact that higher birth weight by itself does not cause OBPI, while it may be associated with certain conditions that cause OBPI. Rather OBPI is more likely caused by the increased forces used in instrumented deliveries [45]. This finding also supports the high correlation with forceps delivery and obpi reported by Foad et al. [1]. 

Our data shows that the SHEAR deformity was significantly more severe in instrument-assisted deliveries, especially when both instruments were used. However, posterior subluxation and glenoid retroversion were not significantly different between spontaneous and instrument-assisted deliveries. This result suggests a mechanism of injury or presence of a risk factor that uniquely affects the development of a SHEAR deformity and is associated with the sequential use of instruments. Shoulder dystocia might be the confounding factor in this case. It has been proposed that the sequential use of instruments is associated with severe shoulder dystocia [31] and brachial plexus injury [32]. It is, however, unlikely that an increase in shoulder dystocia with sequential instrument use can explain these findings because the SHEAR deformity is not more severe in patients with documented shoulder dystocia. Doumouchtsis and Arulkumaran [45] found a strong association between downward traction of the fetal head and OBPI; even without shoulder dystocia they stated that substantial forces were found to be used in many OBPI cases [45]. As traction forces increase, exogenously applied lateral flexion during delivery places force on the brachial plexus [42]. These reports are consistent with our findings, which show that a significant number of our patients suffering from OBPI underwent an instrumented delivery. Nerves in the brachial plexus that are associated with scapular elevation may be considerably more susceptible to injury during instrument assisted deliveries.

It is well established that shoulder dystocia is a significant risk factor for brachial plexus injuries [3]. The shoulder dystocia rate among all brachial plexus injuries in the United States [1], though found to be the most significant risk factor, was reported at a significantly lower rate (18%) than in this study (97%). Our results are similar to a smaller series of permanent OBPP patients which showed a shoulder dystocia rate of 94% [33]. It should be noted that there is no universal definition of shoulder dystocia, hence the variable incidence noted in literature. “True Shoulder Dystocia” was coined by Gross et al. as deliveries requiring, in addition to downward traction and episiotomy, maneuvers to deliver the shoulders [46]. Shoulder dystocia was the most prevalent risk factor in our patients; almost all the children in this study had documented shoulder dystocia and the rate in macrosomic patients was 100%. Shoulder dystocia is, therefore, closely associated with the most severe cases of permanent obstetric brachial plexus injuries.

Lack of finger movement indicates a severe initial injury that extends to C8/T1 and affects the entire brachial plexus. All the muscles in the shoulder are weakened and muscle imbalances are greatly reduced in these injuries compared to C5/C6 injuries that retain finger movement. Severe bony deformities caused by muscle imbalances during a time of rapid growth should thus develop more often in children with finger movement at birth. Posterior subluxation and glenoid retroversion were both significantly less severe in patients with no finger movement at birth. Moreover, the proportion of patients who had no finger movement at birth was higher in non-pseudoglenoid shoulders (68%) than in the pseudoglenoid shoulders (51%). Taken together, the results show that lack of finger movement at birth is actually protective against bony deformities of the shoulder, although the patient suffers extensive functional impairment. Permanently injured patients with finger movement at birth develop more severe bony deformities of the shoulder than patients without finger movement at birth due, in part, to asymmetrical muscle action on developing bony structures.

The strengths of this study are a large sample size (*n* = 241) and a rigorous definition of permanent brachial plexus injury. Risk factors associated with permanent brachial plexus injury should be magnified among patients requiring surgical reconstruction. This study is unique in that it examines the relationship between risk factors at birth and future deformities, as measured by radiographic characteristics. It also determined the value of finger movement at birth, a simple and rapid clinical test, as a predictor of outcome. The birth data in this study is, however, based on retrospective information which was obtained from interviews with patient families. Additionally, a study population of transiently injured patients was not available for comparison.

## Figures and Tables

**Table 1:** Relationship between risk factors at birth and weight among patients with permanent injury.

	Instrument delivery^†^		Shoulder Dystocia		No finger movement	
Birthweight*	%	*n*	%	*n*	%	*n*
All	41	99/239	97	233/241	68	163/241
<3,750 g	47	28/59	88	53/60	65	39/60
3,750–4,499 g	42	55/131	99	131/132	70	92/132
≥4,500 g	33	16/49	100	49/49	66	32/49

*Weight classes were chosen based on Brimacombe et al. [25]: <3750 g: appropriate for gestational age; 3750–4499 g: large for gestational age; ≥4500 g: gross macrosomy.

^†^Includes forceps, vacuum, or both. Information regarding instrument delivery was not available from two patients.

**Table 2:** Risk factors and measurements of osseous deformity.

	PHHA*		Version*		SHEAR*		No finger movement at birth	
*Birth weight*	Avg %	*P*	Avg deg	*P*	Avg %	*P*	%	*P*
>4.5 kg	15 ± 23	0.06	−33 ± 17	0.001	14 ± 16	0.97	65(51–77)	0.82
<4.5 kg	22 ± 21		−26 ± 16		13 ± 11		68(61–74)	

*Delivery*								
Instrument^†^	23 ± 19	0.45	−27 ± 16	0.79	15 ± 12	0.04	69(59–77)	0.91
Spontaneous	20 ± 22		−27 ± 17		11 ± 11		67(59–74)	

*Shoulder Dystocia*								
Yes	20 ± 21	0.61	−28 ± 16	0.25	13 ± 12	0.87	68(62–74)	0.47
No	28 ± 10		−19 ± 6		12 ± 14		50(22–78)	

All patients suffered permanent obstetric brachial plexus injury. Averages are given with standard deviation and were compared with Mann Whitney *U-*test. Proportions were compared with Fisher's exact test and provided with 95% confidence intervals.

*PHHA: percent humeral head anterior to the scapular line. Version: degree the glenoid is rotated from normal (retroversion). SHEAR: scapular hypoplasia, elevation, and rotation as measured by percent scapula superior to clavicle.

^†^Included forceps, vacuum, or both.

**Table 3:** Comparison of osseous deformity in patients with and without finger movement at birth.

	Movement at birth		No movement at birth		
Variable	Avg	*n*	Avg	*n*	* P*
PHHA* (%)	16 ± 20	69	23 ± 22	123	0.005
Version* (deg)	−32 ± 14	69	−25 ± 17	123	0.001
SHEAR* (%)	15 ± 13	58	12 ± 12	94	0.15

	%	*n*	%	*n*	*P*

Non-pseudoglenoid^†^	67 (55–77)	47/70	81 (73–87)	100/124	0.055

All patients suffered permanent obstetric brachial plexus injury. Proportions were compared with Fisher's exact test and provided with 95% confidence intervals. Averages are given with standard deviation and were compared with the Mann Whitney *U-*test.

*PHHA: percent humeral head anterior to the scapular line. Version: degree the glenoid is rotated from normal (retroversion). SHEAR: scapular hypoplasia, elevation, and rotation as measured by percent scapula superior to clavicle.

^†^The glenoid was normal or moderately deformed but had not developed a pseudoglenoid.
